# A Case Report of Moyamoya Disease Presenting as Recurrent Right-Sided Weakness

**DOI:** 10.7759/cureus.39209

**Published:** 2023-05-19

**Authors:** Nabila T Khan, Sumona Islam, Amit Bari

**Affiliations:** 1 Department of Gastroenterology, Delta Hospital, Dhaka, Dhaka, BGD; 2 Department of Gastroenterology, Bangabandhu Sheikh Mujib Medical University, Dhaka, BGD; 3 Department of Nephrology, Kidney Foundation Hospital and Research Institute, Dhaka, BGD

**Keywords:** underdeveloped countries, circle of willis, childhood stroke, intimal thickening and stenosis, moyamoya disease

## Abstract

Moyamoya disease is a cerebrovascular condition characterized by progressive occlusion of the cerebral vessels, particularly in the circle of Willis. This report describes the case of a 12-year-old boy presenting with a history of recurrent right-sided weakness over a period of seven years. Magnetic resonance imaging revealed evidence of both old and recent infarcts, as well as encephalomalacic changes. The diagnosis was confirmed by magnetic resonance angiography, which demonstrated severe stenosis in both internal carotid arteries and the presence of significant collateral formation. In Bangladesh, surgical revascularization for Moyamoya disease had not been previously attempted, and due to financial constraints, the patient’s family opted for conservative management with anti-platelet therapy and regular follow-ups. Although a hereditary component is often presumed in Moyamoya disease, no such familial history was identified in this case. Additionally, no associations with immunological, infectious, hematological, vascular, or congenital syndromes were found. Mortality rates for Moyamoya disease are approximately 10% in adults and 4.3% in children, with a significant proportion of affected individuals experiencing cognitive decline. However, the patient in this case maintained intact cognitive function, and with diligent follow-up and anticoagulation therapy, it was anticipated that his functional capacity would remain stable.

## Introduction

Moyamoya disease is a rare, progressive, steno-occlusive disease of unknown etiology of the cerebral vasculature [1]. Moyamoya disease has the highest incidence rate among Asians, especially in Japan. The male-to-female ratio is approximately 1:1.8. Additionally, the disease is most commonly diagnosed during the first decade of life [2]. Typically, the steno-occlusive areas in Moyamoya disease affect both sides of the brain, although there have been documented cases of unilateral involvement as well [3]. Here, we report the case of a patient with Moyamoya disease who presented to us with repeated right-sided weakness and transient blank staring gaze over a prolonged period of seven years.

## Case presentation

A 12-year-old boy weighing 37 kg presented to us with complaints of repeated weakness in the right side of the body for seven years. The patient’s mother noticed weakness on the right side of her son’s body seven years back, which was sudden in onset and not associated with loss of consciousness, convulsion, fall, vomiting, tongue bite, or bladder-bowel incontinence. He recovered spontaneously after five minutes without any residual weakness. Since then, he had suffered from similar types of attacks always involving the right side about 10-12 times at an interval of two to three months. He consulted several physicians and was treated with anticonvulsants empirically, but the episodes continued. He made a complete recovery each time. Four months back, the patient again developed sudden-onset right-sided weakness, from which he failed to recover.

He had no history of headache, visual impairment, or preceding head trauma. He was the first child of non-consanguineous parents and was born prematurely at 30 weeks via a normal vaginal delivery at the hospital. There are no signs of mental retardation or learning disabilities. During the physical examination, his vital signs and anthropometric measurements were within the normal range. Muscle power on the right side was assessed as 4 out of 5. Additional systemic examinations showed no abnormalities.

**Figure 1:**
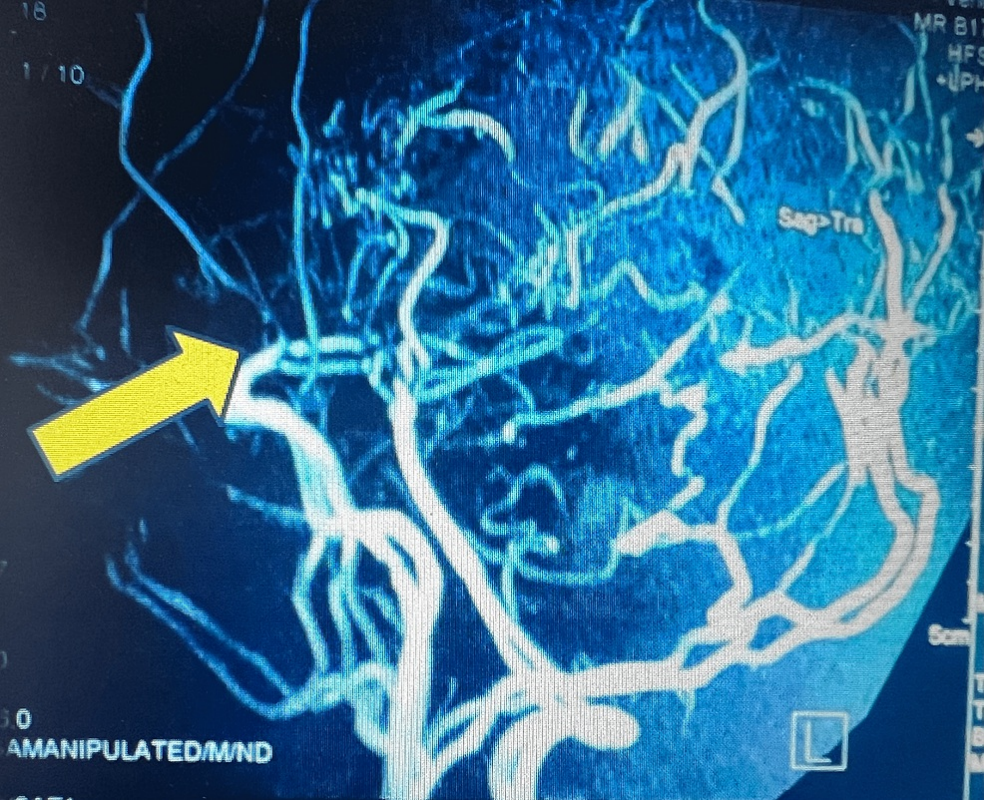
Magnetic resonance angiography revealed severe stenosis at the terminal part of both internal carotid arteries with narrowed middle cerebral artery (MCA) and severe vascular paucity at both MCA territories.

Laboratory investigations revealed a normal complete blood count with erythrocyte sedimentation rate, and metabolic and lipid profile. Prothrombin time and renal function were normal. Anti-nuclear antibodies, anti-phospholipid antibodies, P-antineutrophilic cytoplasmic antibodies (P-ANCA), and C-ANCA were negative. Serum thyroid-stimulating hormone and blood homocysteine levels were also normal. Chest X-ray, electrocardiogram, and echocardiography revealed no abnormalities. Magnetic resonance imaging (MRI) of the brain was suggestive of old infarction with encephalomalacic change in the capsule-ganglionic region of the right cerebral hemisphere and recent infarction in the paraventricular region and centrum semiovale of the left cerebral hemisphere. Electroencephalogram showed interictal epileptiform discharges. Magnetic resonance angiography (MRA) revealed severe stenosis at the terminal part of both internal cerebral arteries with narrowed middle cerebral artery (MCA) and severe vascular paucity at both MCA territories (Figure 1).

Significant collaterals were noted at deep white matter and the thalamo-capsulo ganglionic regions. Magnetic resonance venography was normal.

Considering the associated financial burden, the patient’s family opted for medical management. He was treated conservatively with anti-platelets and regular follow-ups.

## Discussion

Moyamoya disease is a progressive occlusive disease of the cerebral vasculature [3]. There is a presumed hereditary component [4-7]. In this condition, there is a development of intimal thickening in the walls of the terminal segments of both internal carotid arteries. These areas may contain lipid deposits in certain cases. Furthermore, there can be varying levels of narrowing or complete blockage in the anterior, middle, and posterior cerebral arteries that originate from the circle of Willis [1]. Fibrocellular thickening of the intima, which leads to the waving of the internal elastic lamina and thinning of the media, was present [3,8]. However, no familial history indicating a hereditary pattern was discovered.

More often than not Moyamoya disease may occur by itself in a previously healthy individual. It has been associated with various conditions, including immunologic factors such as Grave’s disease, infectious diseases such as leptospirosis and tuberculosis, and hematologic disorders such as aplastic anemia, Fanconi anemia, sickle cell anemia, and lupus anticoagulant. Additionally, vascular factors such as atherosclerotic disease, coarctation of the aorta, fibromuscular dysplasia, cranial trauma, radiation injury, parasellar tumors, and hypertension have been linked to the disease. Moyamoya disease has also been reported in conjunction with congenital syndromes such as Down syndrome, Marfan syndrome, tuberous sclerosis, Turner syndrome, von Recklinghausen disease (neurofibromatosis type 1), and Hirschsprung disease [9-12]. In our case, we searched for such associations with a thorough history and examination, along with relevant and feasible investigations. There was no such associated condition.

The clinical presentations of Moyamoya disease differ between children and adults. The symptoms and course of the disease can range widely from being asymptomatic to presenting with severe neurological deficits [13]. In children, the resulting symptoms may include hemiparesis, monoparesis, sensory impairment, involuntary movements, headaches, dizziness, or seizures. Mental retardation or persistent neurological deficits can also occur [2]. Cerebral ischemic events are more frequently observed in children with Moyamoya disease. Adults, on the other hand, may experience symptoms and signs similar to those seen in children. However, sudden-onset intraventricular, subarachnoid, or intracerebral hemorrhages are more commonly observed in adults [8]. The disease first manifested in our patient at the age of five and followed this usual pattern of presentation, recurrent transient hemiparesis at first, which progressed to become persistent. There may have been associated absent seizures, but no mental retardation.

Digital subtraction angiography (DSA) is a valuable tool for the diagnosis and follow-up of Moyamoya disease. It provides high-resolution images of the cerebral vasculature and allows for the evaluation of the degree of stenosis or occlusion in the affected vessels. DSA also enables the visualization of collateral circulation, which is a hallmark of this disease. Several studies have demonstrated the utility of DSA in the diagnosis and management of Moyamoya disease [14].

Surgical revascularization has emerged as a promising primary treatment option for Moyamoya disease, particularly when medical therapy fails to provide sufficient improvement, and surgical success has been well-documented. There are two primary methods of revascularization used in the treatment of Moyamoya disease. The first is the direct method, which involves creating a connection (anastomosis) between a branch of the external carotid artery, typically the superficial temporal artery, and a cortical artery. The second method is the indirect approach, where vascularized tissue supplied by the external carotid artery, such as the dura, temporalis muscle, or the superficial temporal artery itself, is placed in direct contact with the brain. This placement promotes the growth of new blood vessels, facilitating increased blood supply to the underlying cerebral cortex [13,15,16]. Due to financial constraints, we opted for conservative medical management.

Death due to moyamoya disease is usually from hemorrhage. The outcome and prognosis of the disease depend on how frequently the attacks occur and the extent of hemorrhage. The mortality rates associated with Moyamoya disease are estimated to be around 10% in adults and 4.3% in children [2]. Additionally, more than half of the individuals affected by the disease experience a progressive decline in cognitive function, largely attributed to recurrent strokes [3,16]. Patients who seek treatment at an early stage, while their symptoms are still evolving, generally have a more favorable prognosis compared to those who present with static symptoms, which often indicate a completed stroke [2,3,16]. Although our patient presented late with right-sided hemiparesis, his cognitive function was still intact.

## Conclusions

Moyamoya disease is a rare cause of childhood stroke. While surgical correction is a promising treatment option, it is often not possible in a resource-poor setting due to technical and financial constraints. However, with increased awareness, early detection and prevention of recurrent strokes is possible, which may enable patients to lead a near-normal life.
